# Arthroscopic repair of rotator cuff injury with bioabsorbable suture anchor vs. all-suture anchor: a non-inferiority study

**DOI:** 10.1186/s12891-022-06061-7

**Published:** 2022-12-15

**Authors:** Stefano Di Gennaro, Domenico Lecce, Alessio Tarantino, Mauro De Cupis, Erica Bassetti, Pierpaolo Scarnera, Enrico Ciminello, Vittorio Calvisi

**Affiliations:** 1 Polo Sanitario San Feliciano, Rome, Italy; 2 UNIVAQ MeSVA: Università Degli Studi Dell’Aquila Dipartimento Di Medicina Clinica Sanita Pubblica Scienze Della Vita E Dell’Ambiente, Via Mattia Battistini, 44, 00167 Rome, RM Italy; 3 Department of Orthopaedics and Traumatology, C.T.O. Hospital, Rome, Italy; 4 Department of Radiological, Oncological and Pathological Sciences, La Sapienza” University of Rome, Rome, Italy; 5 Italian Implantable Prostheses Registry, Scientific Secretary of the Presidency, Italian National Institute of Health, Rome, Italy; 6 Department of Statistical Science, La Sapienza” University of Rome, Rome, Italy

**Keywords:** Shoulderarthroscopy, Allsutureanchor, Bioabsorbableanchor, Arthroscopy, Rotatorcuffsurgery

## Abstract

**Background:**

Compare all-suture anchors to traditional anchors through clinical and radiological evaluation at pre-established end-points.

**Materials and methods:**

We performed a two-arms non-inferiority study on all-suture anchor (2.3 iconix™, Stryker) device with respect to traditional anchor (5.5 healix Advance™ BR, Depuy/Mitek) device under unpaired samples with size equal to 30 patients per group, all suffering from supraspinatus tendon rupture. We administrated DASH (Disabilities of the Arm, Shoulder and Hand); constant; and SST (Simple Shoulder Test) questionnaires in pre-operative, 3 ± 1 months post-intervention and 8 ± 1 months post-intervention. Questionnaires scores were the primary outcome. We also evaluated RMI at 3 and at 8 months after surgery to assess the presence of oedema or any loosening of the implant.

**Results:**

All-suture anchor approach has been proven to have non-inferior performances with respect to traditional anchor approach, according to questionnaires scores at the 3-month endpoint. We observed 26 patients with oedema by MRI (18 in control group, 6 in experimental group). In the 8-month endpoint we found persistent edema in 12 patients (all treated with healix), 2 had mobilitazions (healix), 10 had partial retears (8 healix, 2 iconix) and 1 implant failure (healix).

**Conclusions:**

All suture devices have clinical and functional results comparable to traditional devices, while they tend to give fewer complications in terms of bone edema, loosening and retear rate. The effectiveness of all-suture devices should be further investigated in rotator cuff suture arthroscopic revision surgery, given the advantages they offer.

## Introduction

Rotator cuff injury is a widespread pathology in many different types of patients. Its etiology is multifactorial: it can depend on traumatic events, a degenerative process, or a combination of these two factors [1]. Predisposing factors include: age, physically stressful jobs, intense sports activity (especially overhead sports), repeated microtraumatisms, metabolic diseases [2]. The most affected area by these injuries is the insertional portion of the tendon, due to its mechanical characteristics and the greater mechanical effort to which this area is subjected. Currently, international literature suggests that the best strategy for repairing rotator cuff lesions is arthroscopic surgery using suture anchors [3]. Recently, a new all-suture anchor has been developed. This device has theoretical advantages compared to traditional anchors, as shown in some biomechanical studies on cadavers, which have shown a reduction in the effects of pullout and less invasiveness on tissues, with lesions of the bone tissue and areas of bone defect considerably reduced in case of pullout, a very relevant factor especially in case of need for reoperation [4]. Indeed, we believe that the use of such all-suture anchor, given its less invasive nature, may be preferable in the surgical treatment of young patients or patients at greater risk of reoperation, due to the significant reduced trauma that these devices apply to the tissues.

Therefore, aim of this study is to test the non-inferiority of all-suture devices with respect to traditional ones, by investigation of clinical and radiological outcomes in arthroscopic rotator cuff repair surgery. We compared two different types of anchors: a standard bioabsorbable threaded suture anchor (5.5 healix Advance™ BR, Depuy/Mitek) and an all-suture anchor (2.3 iconix™, Stryker) by evaluating clinical and radiological results [5].

## Materials and methods

The performance of the two types of anchors was evaluated via the administration of three validated questionnaires to the patients, namely: DASH (Disabilities of the Arm, Shoulder and Hand); constant; and SST (Simple Shoulder Test) [6, 7]. The questionnaires were administrated by the clinicians to the patients in three different moments: pre-operative (time 0), 3 ± 1 months post-intervention (time 1) and 8 ± 1 months post-intervention (time 2), in order to investigate the possible functional improvement of the patients, highlighted by changes in the resulting scores.

### Patients enrollment and statistical analysis

A two arms non-inferiority study was performed, where the performance of 2.3 iconix™, Stryker, used in experimental treatment group (referred to also as iconix group in the following), was tested against the performance of 5.5 healix Advance™ BR, Depuy/Mite, used in control group (referred to also as healix group in the following).

The main outcome and measure of comparison between treatments was the difference in terms of the average DASH score between the two groups of patients**.** The differences in terms of the average constant and SST scores between the two groups of patients were the secondary outcomes**.** The sample size was determined equal to 30 patients in each group, while considering a 0.8 power for the test with 0.95 significance and by fixing sd = 26 and $\partial$= 12 in the following non-inferiority test:

$$\left\{\begin{array}{c}{H}_{0}: E-C \le -\partial \\ {H}_{1}: E-C > -\partial ,\end{array}\right.$$

where *E* is outcome in terms of DASH score of the experimental treatment and *C* is the outcome of the control group treatment. The considered value of the sd is the first integer to guarantee a 95% confidence interval length equal to 100 on a normal distribution; the fixed value for $\partial$ is given by a integer approximation of the quantity identified by van Kampen and co-authors as Minimal Important Change in shoulder-related PROMs when comparing performance of devices between groups in terms of DASH score [8].

Between February 2016 and May 2017, 60 patients with total rupture of the supraspinatus tendon were enrolled in the study according to the following inclusion criteria, that are the same for the two groups: over than 40 years of age; no previous surgery on the same shoulder; absence of comorbidities of the long head of the biceps that involve tenotomy/tenodesis; absence of concomitant lesion of other rotator cuff tendons; no neoplastic pathologies.

Continuous variables are presented in terms of *average(sd)*, while categorical variables are presented in terms of *absolute frequencies(percentage in the group)*. The significance of the differences between the groups are tested by t-test for continuous variables and by ${\chi }^{2}$ test for categorical variables, with significance level for the p-value fixed equal to 0.05.

The resulting score for the three considered questionnaires (DASH, constant, SST) at 3-months check-ups were compared between the two groups to investigate statistically significant differences by unilateral t-tests for unpaired groups with significance level at 0.05. The tests were performed taking into account that: for the DASH scale, a lower score means a better clinical outcome; for constant and SST scales, a higher score means a better clinical performance.

The statistical analysis was performed by using the software R version 3.6.3 (2020–02-29) – "Holding the Windsock".

### Surgical technique

Surgery was performed by a skilled surgeon with more than 20 years of experience in accordance with the principles established and recognized by international literature [9, 10]. The torn supraspinatus tendon was repaired arthroscopically using 1 or more anchors and the same knotting technique. Surgical access was performed through standard arthroscopic portals. Patients underwent intraoperative assessment of the lesion (extent, tissue quality, reducibility of the lesion). Repair was performed after debridement of the lesion and the footprint, with reinsertion on the bone. At the end of the procedure all patients underwent minimal acromioplasty, not for biomechanical purposes but with the aim of providing regenerative input.

All patients underwent arthroscopic surgery, followed by a 2-day hospital stay, with the instruction to wear a 15° abduction brace for 3 weeks and observe the same standardized rehabilitation protocol.

All the patients underwent tenotomy of the long head of the biceps during the arthroscopic procedure due to its irreparable lesion.

The quality of the tendon tissue was assessed intraoperatively by testing its consistency, elasticity and mechanical strength.

### Radiological evaluation

Patients underwent clinical and radiological (MR) assessments at time 0, time 1 and time 2. The presence of pain was assessed by the VAS scale at time 0, 1 and 2. MR evaluation at time 1 was intended only to verify possible bone edema, while the time 2 check-up aimed to ascertain the presence of bone edema, mobilization of the anchors, suture and tendon status. The time 1 and 2 MR exam was accomplished through the use of sequences suitable for evaluating the region of interest; in particular, a 0.2 T Opera Esaote magnet was used. Images with a 13 cm FOV (Field Of View) were acquired, with a layer thickness of 5 mm. Routine sequences were Spin Echo T1 weighted, Turbo-Spin Echo T2 weighted and STIR sequences in the coronal scan planes, Spin Echo T1 weighted sequences and Turbo-Spin Echo T2 in the axial scan planes and Spin Echo T1 sequences weighted in the sagittal planes.

The diagnostic tests were performed in accordance with the ethical standards of the Evaluation Committee on Human Experimentation and with the Helsinki Declaration of 1975, revised in 2008. Informed consent was obtained from all patients who participated in the study.

## Results

The sample consisted of 32 females and 28 males. Comorbidities were observed in 26 patients: 22 patients with high blood pressure (13 healix, 9 iconix) and 4 with diabetes mellitus (1 healix, 3 iconix). The tendon lesion affected the dominant shoulder in 42 patients (14 healix, 18 iconix). Every-day activities of 12 patients (4 healix, 8 iconix) were characterized by the risk of shoulder wear and tear, while 26 (12 healix, 14 iconix) were engaged in sports associated with a tendon injury. All patients were affected by lesions greater than one centimeter in length. Moderate tissue quality was detected in 36 patients (20 healix, 16 iconix), while 24 patients presented tissue of poor quality (13 healix, 11 iconix).

Distributions of patients’ characteristics along experimental and control groups were statistically tested and are resumed in Table 1. Statistical tests ensure that the observed differences in pre-operative conditions between the two groups were not statistically significant, that is, the bias in distributions of patients’ features along the two groups is negligible.

**Table 1:** Patients’ features in the two observed groups

	**Healix**	**Iconix**	***p*-value**
Age	63.2 (8.66)	58.93 (9.72)	0.0778
Male	10 (35.71%)	18 (64.29%)	0.0701
Sport Practice	12 (46.15%)	14 (53.85%)	0.7945
Poor tissue quality	10 (41.67%)	14 (58.33%)	0.4292
Starting DASH	99 (21.56)	91.93 (17.81)	0.1716
Starting CONSTANT	61.13 (13.53)	64.6 (19.14)	0.4212
Starting SST	4.8 (2.52)	5.2 (1.94)	0.4938
Edema	24 (66.67%)	12 (33.33%)	0.0037

Results of the functional scores at different endpoints are summarized in Table 2 and overall, across all endpoints considered and with all rating scales used, patients treated with iconix showed better results than patients in the healix group. However, these results did not improve significantly quality of life or functional autonomy, in accordance with the design of the study which aimed to highlight a non-inferiority of iconix compared to healix.

**Table 2:** Scores at endpoints

Score	Time	Healix	Iconix
**DASH**	0	99 (21.56)	91.93 (17.81)
	3 months	80.47 (22.59)	69 (19.14)
	8 months	66.67 (22.38)	56.07 (19.55)
**CONSTANT**	0	61.13 (13.53)	64.6 (19.14)
	3 months	74.07 (11.71)	79.27 (13.37)
	8 months	83.27 (8.22)	89.33 (7.54)
**SST**	0	4.8 (2.52)	5.2 (1.94)
	3 months	6.53 (1.74)	7.27 (2.69)
	8 months	7.87 (2.16)	8.8 (2.14)

**Fig. 1:**
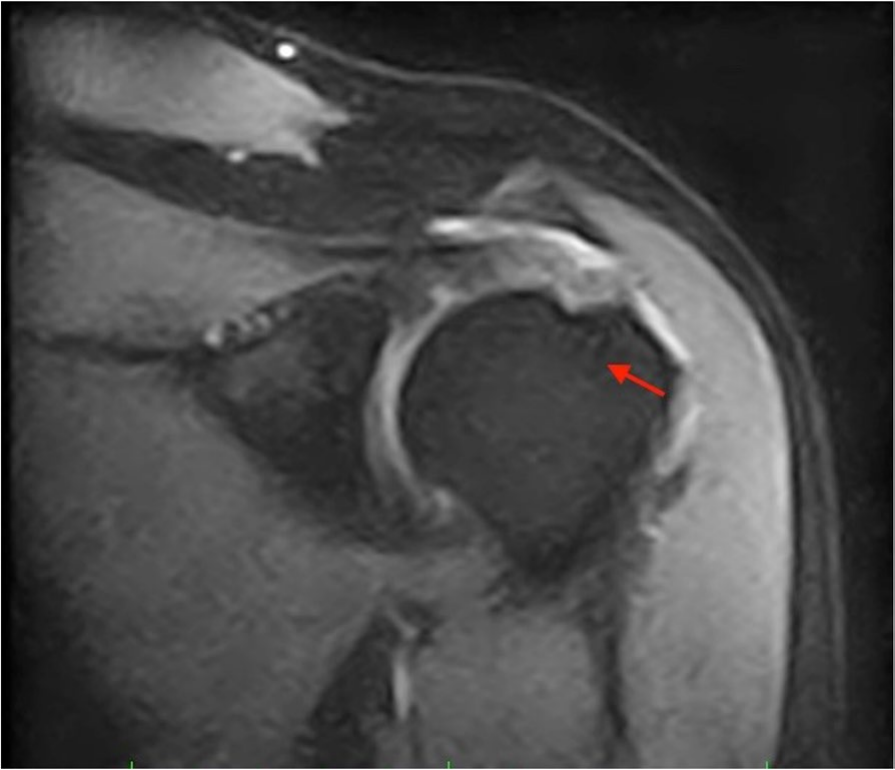
pre-surgery MRI

**Fig. 2:**
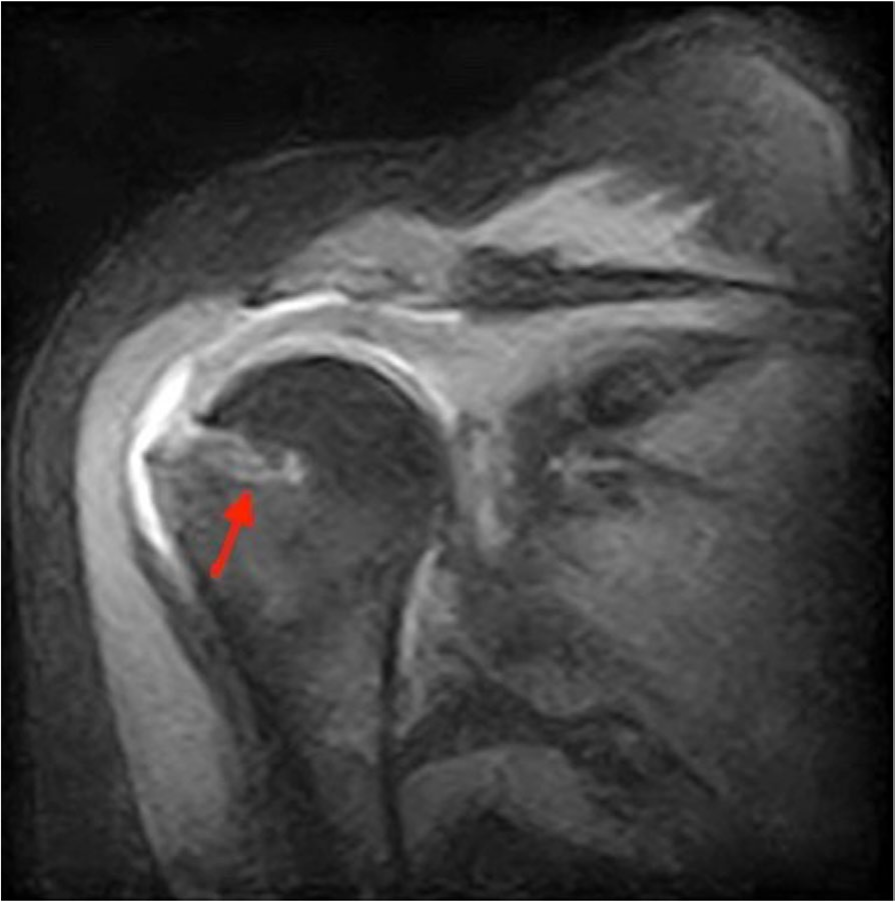
post-surgery MRI

The results of the non-inferiority test for all the considered outcomes at Time 1 show non-inferiority of the iconix anchor (Table 3), according to one-sided t-test and this is statistically significant for DASH, constant and SST scores (*p* < 0.0001), while considering $\partial$= 12, as fixed in the study design. At the 3-month check-up (Time 1) with MRI [Figs. 1 and 2], intraosseous edema was detected in 26 patients (18 healix, 8 iconix). After 8 months (Time 2) it was possible to observe a reduction in edema, with 12 cases in all (12 healix, no edema in iconix). Furthermore, the mobilization of the anchors was detected at time 2 only for two patients in healix group, who presented a partial dislocation, defined as mobilization of less than 2 mm with no signs of suture failure. We also recorded 10 partial retears (8 healix, 2 iconix) and a single case of suture failure (healix).

**Table 3:** Result of the non-inferiority test between the two groups with $\partial$ = 12

	**Δ Iconix vs. Healix**	**95% confidence interval**	***p*-value**
DASH	-11.47	-Inf; -2.43	0.0191
CONSTANT	5.2	-0.22; Inf	0.0572
SST	0.73	-0.24; Inf	0.1074

The assessing for pain by VAS scale showed an average of 2.21(2.18) in experimental group (moderate non-disabling pain), and 4.14(2.17) for control group (*p* = 0.0016).

## Discussion

Rotator cuff injury is a disease that has historically been approached with various therapeutic possibilities: open surgery, conservative therapy and, more recently, arthroscopic repair [11]. Technological innovation and the development of arthroscopic techniques have allowed a progressive expansion of the treatable lesions and have given a significant input to the development of new devices, especially anchors [12]. In our study in particular we used the iconix anchor, characterized by the "all-suture" system, a device smaller in size compared to the other bioabsorbable anchors (healix) and with the possibility of a less demolishing approach on the bone and on the tissues. This technique has already been studied in the literature [13–15] with biomechanical analyses in studies on cadavers [16] and on guinea pigs [17], without however being able to obtain medium-long-term results regarding functional outcomes. All-suture anchors were also tested, again in cadaveric studies, in tenodesis of the long head of the biceps, with encouraging results [18]. In a single study, the functional outcome was assessed by means of constant on a small number of patients, without however a radiological evaluation or a comparison with a similar system used by the same surgeon [19]. It is fair to say that the literature does not provide reliable clinical or instrumental parameters for the prognosis of arthroscopic repair, thus highlighting poor reproducibility of the evaluation indices considered [20, 21].

In our study, we assessed the non-inferiority of the 2.3 iconix™, Stryker "all-suture" system with respect to the 5.5 healix Advance™ BR, Depuy/Mite bioadsorbable anchors. We performed a two-arms non-inferiority study with not paired samples with Dash, constant and SST scores as measures to compare the performance between the treatments. According to the statistical tests, there was not any significant bias in distributions of patients’ features between the two groups. The only observed significant difference is in oedema, that was mostly found in the group treated by the use of the healix anchor, but no evidence for correlation between presence of oedema and post-operative outcome, up to our knowledge, has been found in literature [22]. The results of the study allow us to conclude that the null hypothesis H0 can be rejected and, therefore, the performance of the experimental treatment is at least not lower than the performance of the control one.

Furthermore, we observed a reduced incidence of edema in patients treated with all-suture anchors. However, we did not note any relationship between the dislocations (measured by MR) and the functional results obtained and the patients’ quality of life, thus confirming the thesis expressed by S. H. Kim et al. [23], regarding the non-correlation between the presence of perianchor edema and the functional result of the repair. We also found no significant differences from the results obtained for patients treated with the healix system.

Moreover, pain assessment by VAS scale showed a statistically significant better recovery for patients treated with all-suture anchor device at 8 ± 1 months after the intervention. Considering also what we have seen in the literature, we believe that the best result in terms of pain of the all-sutures is to be attributed to the minor bone trauma compared to the role of bone edema, which the evidence seems to suggest is negligible.

Lastly, the less invasive nature of all-suture systems on the bone must be recognized: this is a factor which has been proved as extremely important in a disease that often occurs in an active population with a significant incidence of retear [24]. In fact, studies on cadavers have shown that all-suture anchors can be removed in revision surgery and allow the implant site to be used as a new footprint for traditional revision, thus making any reintervention on a cuff rupture technically less difficult [25].

The study has several limitations. The considered sample is small and the follow-up is limited to 2 controls within 1 year from surgery. However, there are not yet any clinical studies that have investigated all-suture devices on rotator cuff arthroscopic repair and the considered outcomes suggest an optimal or satisfactory recovery that makes a longer follow-up less likely to invalidate the obtained results.

## Conclusion

In our experience, an all-suture system offers results comparable to the ones obtained in patients treated with traditional anchors, with the advantage of a small number of edema cases and tendon retears, as well as mechanical failure is less likely to be observed. Further studies, preferably randomized and multi-center, would provide more case histories, as well as extending the endpoints, and would verify any long-term outcomes observed in patients treated with iconix anchors.

## Data Availability

Data are available from the corresponding author upon request.

## Abbrevations

MR: Magnetic resonance
DASH: Disabilities of the Arm, Shoulder and Hand
SST: Simple Shoulder Test

## References

[1] Seitz AL, McClure PW, Finucane S, Boardman ND, Michener LA (2011). Mechanisms of rotator cuff tendinopathy: intrinsic, extrinsic, or both?. Clin Biomech.

[2] Leong HT, Fu SC, He X, Oh JH, Yamamoto N, Yung SHP (2019). Risk factors for rotator cuff tendinopathy: a systematic review and meta-analysis. J Rehabil Med.

[3] Huegel J, Williams AA, Soslowsky LJ (2014). Rotator cuff biology and biomechanics: a review of normal and pathological conditions. Curr Rheumatol Rep.

[4] Ntalos D, Huber G, Sellenschloh K, Saito H, Püschel K, Morlock MM, Frosch KH, Klatte TO (2021). All-suture anchor pullout results in decreased bone damage and depends on cortical thickness. Knee Surg Sports Traumatol Arthrosc.

[5] Papalia R, Franceschi F, Diaz Balzani L, D’Adamio S, Denaro V, Maffulli N (2014). The arthroscopic treatment of shoulder instability: Bioabsorbable and standard metallic anchors produce equivalent clinical results. Arthroscopy.

[6] Angst F, Schwyzer HK, Aeschlimann A, Simmen BR, Goldhahn J (2011). Measures of adult shoulder function: Disabilities of the Arm, Shoulder, and Hand Questionnaire (DASH) and Its Short Version (QuickDASH), Shoulder Pain and Disability Index (SPADI), American Shoulder and Elbow Surgeons (ASES) Society Standardized Shoulder Assessment Form, Constant (Murley) Score (CS), Simple Shoulder Test (SST), Oxford Shoulder Score (OSS), Shoulder Disability Questionnaire. Arthritis Care Res.

[7] Carosi M, Galeoto G, Di Gennaro S, Berardi A, Valente D, Servadio A. Transcultural reliability and validity of an Italian language version of the constant–Murley score. J Orthop Trauma Rehab. 2020;221049172094532. 10.1177/2210491720945327

[8] van Kampen DA, Willems WJ, van Beers LW, Castelein RM, Scholtes VA, Terwee CB (2013). Determination and comparison of the smallest detectable change (SDC) and the minimal important change (MIC) of four-shoulder patient-reported outcome measures (PROMs). J Orthop Surg Res.

[9] Farmer KW, Wright TW (2015). Shoulder arthroscopy: the basics. J Hand Surg Am.

[10] Paxton ES, Backus J, Keener J, Brophy RH (2013). Shoulder arthroscopy: basic principles of positioning, anesthesia, and portal anatomy. J Am Acad Orthop Surg.

[11] Dang A, Davies M (2018). Rotator Cuff Disease: Treatment Options and Considerations. Sports Med Arthrosc Rev.

[12] Visscher LE, Jeffery C, Gilmour T, Anderson L, Couzens G (2019). The history of suture anchors in orthopaedic surgery. Clin Biomech.

[13] Byrd JWT, Jones KS, Loring CL, Sparks SL (2018). Acetabular all-suture anchor for labral repair: incidence of intraoperative failure due to pullout. Arthroscopy.

[14] Lacheta L, Dekker TJ, Anderson N, Goldenberg B, Millett PJ (2019). Arthroscopic knotless, tensionable all-suture anchor bankart repair. Arthrosc Tech.

[15] Lee JH, Park I, Hyun HS, Kim SW, Shin SJ (2019). Comparison of clinical outcomes and computed tomography analysis for tunnel diameter after arthroscopic Bankart repair with the all-suture anchor and the biodegradable suture anchor. Arthroscopy.

[16] Nagra NS, Zargar N, Smith RDJ, Carr AJ (2017). Mechanical properties of all-suture anchors for rotator cuff repair. Bone Joint Res.

[17] Barber FA, Herbert MA (2013). Cyclic loading biomechanical analysis of the pullout strengths of rotator cuff and glenoid anchors: 2013 update. Arthroscopy.

[18] Frank RM, Bernardoni ED, Veera SS, Waterman BR, Griffin JW, Shewman EF, Verma NN (2019). Biomechanical analysis of all-suture suture anchor fixation compared with conventional suture anchors and interference screws for biceps tenodesis. Arthroscopy.

[19] Dhinsa BS, Bhamra JS, Aramberri-Gutierrez M, Kochhar T (2019). Mid-term clinical outcome following rotator cuff repair using all-suture anchors. J Clin Orthop Trauma.

[20] Saccomanno MF, Cazzato G, Fodale M, Sircana G, Milano G (2015). Magnetic resonance imaging criteria for the assessment of the rotator cuff after repair: a systematic review. Knee Surg Sports Traumatol Arthrosc.

[21] Saccomanno MF, Sircana G, Cazzato G, Donati F, Randelli P, Milano G (2016). Prognostic factors influencing the outcome of rotator cuff repair: a systematic review. Knee Surg Sports Traumatol Arthrosc.

[22] Chen S, He Y, Wu D, Hu N, Liang X, Jiang D, Huang W, Chen H (2021). Postoperative bone marrow edema lasts no more than 6 months after uncomplicated arthroscopic double-row rotator cuff repair with PEEK anchors. Knee Surg Sports Traumatol Arthrosc.

[23] Kim SH, Yang SH, Rhee SM, Lee KJ, Kim HS, Oh JH (2019). The formation of perianchor fluid associated with various suture anchors used in rotator cuff repair: all-suture, polyetheretherketone, and biocomposite anchors. Bone Joint J.

[24] Lee YS, Jeong JY, Park CD, Kang SG, Yoo JC (2017). Evaluation of the risk factors for a rotator cuff retear after repair surgery. Am J Sports Med.

[25] Ntalos D, Huber G, Sellenschloh K, Briem D, Püschel K, Morlock MM, Klatte TO (2019). Biomechanical analysis of conventional anchor revision after all-suture anchor pullout: a human cadaveric shoulder model. J Shoulder Elbow Surg.

